# Resistance to IL-10 inhibition of interferon gamma production and expression of suppressor of cytokine signaling 1 in CD4^+ ^T cells from patients with rheumatoid arthritis

**DOI:** 10.1186/ar1445

**Published:** 2004-10-13

**Authors:** Jiro Yamana, Masahiro Yamamura, Akira Okamoto, Tetsushi Aita, Mitsuhiro Iwahashi, Katsue Sunahori, Hirofumi Makino

**Affiliations:** 1 Department of Medicine and Clinical Science, Graduate School of Medicine and Dentistry, Okayama University, Okayama, Japan

**Keywords:** CD4^+ ^T cells, IL-10, rheumatoid arthritis, signal transducer and activator of transcription 3, suppressor of cytokine signaling 1

## Abstract

IL-10 has been shown to block the antigen-specific T-cell cytokine response by inhibiting the CD28 signaling pathway. We found that peripheral blood CD4^+ ^T cells from patients with active rheumatoid arthritis (RA) were able to produce greater amounts of interferon gamma after CD3 and CD28 costimulation in the presence of 1 ng/ml IL-10 than were normal control CD4^+ ^T cells, although their surface expression of the type 1 IL-10 receptor was increased. The phosphorylation of signal transducer and activator of transcription 3 was sustained in both blood and synovial tissue CD4^+ ^T cells of RA, but it was not augmented by the presence of 1 ng/ml IL-10. Sera from RA patients induced signal transducer and activator of transcription 3 phosphorylation in normal CD4^+ ^T cells, which was mostly abolished by neutralizing anti-IL-6 antibody. Preincubation of normal CD4^+ ^T cells with IL-6 reduced IL-10-mediated inhibition of interferon gamma production. Blood CD4^+ ^T cells from RA patients contained higher levels of suppressor of cytokine signaling 1 but lower levels of suppressor of cytokine signaling 3 mRNA compared with control CD4^+ ^T cells, as determined by real-time PCR. These results indicate that RA CD4^+ ^T cells become resistant to the immunosuppressive effect of IL-10 before migration into synovial tissue, and this impaired IL-10 signaling may be associated with sustained signal transducer and activator of transcription 3 activation and suppressor of cytokine signaling 1 induction.

## Introduction

IL-10 is a key cytokine in regulating inflammatory responses, mainly by inhibiting the production and function of proinflammatory cytokines. IL-10 binds to the IL-10 receptor (IL-10R) complex that is composed of two subunits, the primary ligand-binding component type 1 IL-10R (IL-10R1) and the accessory component type 2 IL-10R [1]. The interaction of IL-10 and IL-10R engages the Janus kinase (JAK) family tyrosine kinases Jak1 and Tyk2, which are constitutively associated with IL-10R1 and type 2 IL-10R, respectively [2]. IL-10 induces tyrosine phosphorylation and activation of the latent transcriptional factors signal transducer and activator of transcription (STAT) 3 and STAT1 [3]. Upon phosphorylation, STAT1 and STAT3 proteins form homodimers or heterodimers, rapidly translocate into the nucleus, and modulate gene transcription. Intriguingly, STAT3 is indispensable for both IL-10-derived anti-inflammatory and IL-6-derived proinflammatory responses [4]. Studies of cell-type-specific STAT3-deficient mice have shown that STAT3 activation is essential for IL-10-mediated anti-inflammatory reactions in macrophages and neutrophils [5], but is responsible for IL-6-mediated prevention of apoptosis in T cells [6]. The suppressor of cytokine signaling (SOCS) proteins have been identified as a family of endogenous JAK kinase inhibitors that can act in classic feedback inhibition loops, but their roles as the mediators of crosstalk inhibition by opposing cytokine signaling pathways have been clarified [7]. Recent studies indicate that SOCS3 plays a key role in regulating the divergent action of IL-10 and IL-6, by specifically blocking STAT3 activation induced by IL-6 but not that induced by IL-10 [8,9].

The synovial membrane of rheumatoid arthritis (RA) is characterized by an infiltrate of a variety of inflammatory cells, such as lymphocytes, macrophages, and dendritic cells, together with proliferation of synovial fibroblast-like cells. Numerous cytokines are overproduced in the inflamed joint, and macrophages and synovial fibroblasts are an important source of proinflammatory cytokines. Tumor necrosis factor alpha (TNF-α) and IL-1, two major macrophage products, are crucial in the process of chronic inflammation and joint destruction, and they give rise to effector components, including other inflammatory cytokines, chemokines, growth factors, matrix proteases, nitric oxide, and reactive oxygen species [10]. IL-6 is a pleiotropic cytokine produced substantially by activated fibroblasts, and its proinflammatory actions include simulating the acute-phase response, B-cell maturation into plasma cells, T-cell functions, and hematopoietic precursor cell differentiation [11].

However, anti-inflammatory cytokines and cytokine inhibitors are also present in large quantities in RA joints. IL-10, produced by macrophages and partly by T cells in the synovial tissue (ST), is best known as a negative regulator for macrophage and Th1 cells, but the expression level is insufficient to counterbalance the cascade of proinflammatory events [12]. In addition, the anti-inflammatory action of IL-10 appears to be modulated at the level of signal transduction during chronic inflammation. IL-10 signaling is impaired in macrophages upon chronic exposure to proinflammatory cytokines such as TNF-α and IL-1 and immune complexes [13,14]. Cell surface expression of IL-10R1 is decreased in synovial fluid dendritic cells due to the presence of TNF-α, IL-1, and granulocyte–macrophage colony-stimulating factor [15].

CD4^+ ^T cells may be activated by arthritogenic antigens, in conjunction with CD28-mediated costimulatory signaling, in RA. The significance of this autoimmune process has been supported by the linkage of the MHC class II antigens HLA-DRB1*0404 and HLA-DRB1*0401 with disease susceptibility and severity [16,17], and by the high-level expression of MHC class II molecules and both CD28 ligands, CD80 and CD86, in the inflamed ST [18-20]. The continuing emergence of activated CD4^+ ^T cells, even though few in number, may be crucial in sustaining the activation of macrophages and synovial fibroblasts through cell surface signaling by means of cell surface CD69 and CD11, as well as the release of proinflammatory Th1 cytokines such as interferon gamma (IFN)-γ and IL-17 [21,22]. In addition, CD4^+ ^T cells could stimulate B-cell production of autoantibodies such as rheumatoid factor and osteoclast-mediated bone destruction. Their obligatory role in RA synovitis was recently proved by successful treatment of active disease by selective inhibition of T-cell activation with fusion protein of cytotoxic T-cell-associated antigen 4 (CD152)-IgG, which can block the engagement of CD28 on T cells by binding to CD80 and CD86 with high avidity [23].

IL-10 efficiently blocks the antigen-specific T-cell cytokine response by inhibiting the CD28 signaling pathway [24], as well as indirectly by downregulating the function of antigen-presenting cells. To elucidate the resistance of CD4^+ ^T cells to this direct inhibition in RA, we investigated the production of IFN-γ after CD3 and CD28 costimulation in the presence of IL-10, the induction of STAT1 and STAT3 phosphorylation by IL-10, and the expression of SOCS1 and SOCS3 mRNA in peripheral blood (PB) CD4^+ ^T cells from RA patients.

## Materials and methods

### Patients and samples

The total patient population consisted of 32 patients with RA (25 women and seven men; mean ± standard deviation age, 52.8 ± 12.4 years) diagnosed according to the revised 1987 criteria of the American College of Rheumatology (formally, the American Rheumatism Association) [25]. All patients were receiving prednisolone (≤ 7.5 mg/day) and disease-modifying antirheumatic drugs. Clinical parameters in the study patients were as follows (mean ± standard deviation): erythrocyte sedimentation rate, 55.9 ± 35.4 mm/hour; serum C-reactive protein (CRP) level, 32.0 ± 32.0 mg/l; and IgM class rheumatoid factor titer, 142 ± 158 U/ml. Patients were divided into two groups: 24 patients with active disease, who had multiple tender and/or swollen joints and elevated serum CRP level (≥ 10 mg/l); and eight patients with inactive disease, who satisfied the American College of Rheumatology preliminary criteria for clinical remission [26]. Sixteen healthy volunteers (11 women and five men; age, 45.8 ± 11.2 years) served as controls. ST samples were obtained from three RA patients undergoing total knee replacement. All patients gave informed consent.

### Isolation of CD4^+ ^T cells

Peripheral blood mononuclear cells (PBMC) were prepared from heparinized blood samples by centrifugation over Ficoll-Hypaque density gradients (Pharmacia, Uppsala, Sweden). CD4^+ ^T cells were purified from PBMC by positive selection using anti-CD4 mAb-coated magnetic beads (Miltenyi Biotec, Gladbach, Germany), according to the manufacturer's instructions. CD4^+ ^T cells were isolated from ST samples, as previously described [27]. Briefly, fresh ST samples were fragmented and digested with collagenase and DNase for 1 hour at 37°C. After removing tissue debris, ST cell suspensions in culture medium (RPMI 1640 medium; Life Technologies, Gaithersburg, MD, USA) supplemented with 25 mM HEPES (2 mM L-glutamine, 2% nonessential amino acids, 100 IU/ml penicillin, and 100 mg/ml streptomycin; Life Technologies) with 10% heat-inactivated FCS (Life Technologies) were incubated at 37°C in six-well plates (Coster, Cambridge, MA, USA) for 45 min. Non-adherent cells were harvested and CD4^+ ^T cells were purified by positive selection as already described.

### Culture of CD4^+ ^T cells

PB CD4^+ ^T-cell populations were resuspended at a density of 1 × 10^6 ^cells/ml in culture medium with 10% FCS, and 0.5 ml cell suspensions were dispensed into the wells of 24-well microtiter plates (Coster) coated with 1 μg/ml anti-CD3 mAb (Immunotech, Marseille, France). The cells were incubated with 1 μg/ml anti-CD28 mAb (Immunotech) in the presence or absence of the indicated concentrations of IL-10 (Becton Dickinson, San Jose, CA, USA) at 37°C in a humidified atmosphere containing 5% CO_2 _[28]. Culture supernatants were collected 36 hours later and cell-free samples were stored at -30°C until cytokine assay.

To examine the effect of IL-6 on T-cell responsiveness to IL-10, CD4^+ ^T cells from healthy controls were incubated in culture medium with 10% FCS in the presence or absence of 10 ng/ml IL-6 (Becton Dickinson) for 36 hours. Cells were then stimulated for 36 hours with anti-CD3 mAb and anti-CD28 mAb in the presence or absence of 1 ng/ml IL-10. Culture supernatants were measured for IFN-γ concentrations.

### Flow cytometric analysis for IL-10R1 expression

A sample of 5 × 10^5 ^cells of PBMC was resuspended in PBS with 1% FCS. PBMC were incubated with saturating concentrations of anti-IL-10R1 mAb (IgG_1_; R&D systems, Minneapolis, MN, USA) or with isotype-matched control mAb (Immunotech), followed by incubation with FITC-conjugated goat anti-mouse IgG_1 _polyclonal antibody (Santa Cruz Biotechnologies, Santa Cruz, CA, USA). Cells were then incubated with phycoerythrin-conjugated anti-CD4 mAb (Becton Dickinson). Cells were washed well with 1% FCS/PBS between incubations. Analysis was performed on a FACScan flow cytometer (Becton Dickinson).

### Immunoassay for IFN-γ and IL-2

Concentrations of IFN-γ and IL-2 in culture supernatants of CD4^+ ^T cells were measured in duplicate by the quantitative sandwich ELISA using cytokine-specific capture with biotinylated detection mAb and recombinant cytokine proteins (all from Becton Dickinson), according to the manufacturer's protocol. The detection limits for IFN-γ and IL-2 were 15 pg/ml.

### Isolation of mRNA and real-time PCR

Total cellular RNA was extracted from PB CD4^+ ^T cells using an RNA isolation kit (RNeasy Mini kit; Qiagen, Valencia, CA, USA), according to the manufacturer's instructions. cDNA was synthesized from total RNA with Molony murine leukemia virus reverse transcriptase (US Biochemical, Cleveland, OH, USA) and oligo-(dT)_15 _primers (Promega, Madison, WI, USA). Real-time PCR was performed with the LightCycler Instrument (Roche Diagnostics, Penzberg, Germany) in glass capillaries. The reaction mix containing Taq DNA polymerase and DNA double-strand-specific SYBR Green I dye (Lightcycler FastStart DNA Master SYBR Green I; Roche Diagnostics) and specific primers were added to cDNA dilutions.

The cDNA samples were denatured at 95° C for 10 min, and were then amplified for 40–50 cycles: at 95° C (10 s), at 65° C (15 s), and 72° C (22 s) for β-actin; at 95° C (10 s), at 62° C (15 s), and at 72° C (10 s) for SOCS1; and at 96° C (10 s), at 68° C (15 s), and at 72° C (15 s) for SOCS3. Amplification curves of the fluorescence values versus cycle number were obtained, and a melting curve analysis was then performed. The levels of SOCS1 and SOCS3 expression were determined by normalizing relative to β-actin expression. The forward and reverse primers were as follows: for β-actin, 5'-GTGGGGCGCCCCAGGCACCA-3' and 5'-CTCCTTAATGTCACGCACGATTTC-3' ; for SOCS1, 5'-AGACCCCTTCTCACCTCTTG-3' and 5'-GCACAGCAGAAAAATAAAGC-3' ; and for SOCS3, 5'-CCCGCCGGCACCTTTCTG-3' and 5'-AGGGGCCGGCTCAACACC-3'.

### Western blot analysis

CD4^+ ^T cells were stimulated for 20 min by the indicated concentrations of IL-10 and IL-6 at a density of 5 × 10^5 ^cells in 0.5 ml culture medium with 10% FCS. To examine the effect of serum IL-6 on STAT phosphorylation, normal CD4^+ ^T cells were stimulated for 20 min with 30% active RA serum in culture medium with 40 μg/ml neutralizing goat anti-IL-6 polyclonal antibody (IgG; Techne, Princeton, NJ, USA) or control goat IgG (Techne). Whole cell lysates were prepared by placing cells in 100 μl SDS lysing buffer (62.5 mM Tris–HCl [pH 6.8], 2% SDS, 10% glycerol, 50 mM dithiothreitol, 0.1% bromphenol blue). Then 20 μl protein samples were fractionated on 10% SDS-polyacrylamide gels and were transferred to nitrocellulose membranes (Amersham, Buckinghamshire, UK), and the membrane was blocked with 5% skim milk in Tris-buffered saline with 0.1% Tween 20.

Tyrosine phosphorylation of STAT1 and STAT3 was detected using commercial available kits (Cell Signaling Technology, Beverly, MA, USA) according to the manufacturer's instructions. Briefly, the membrane was incubated with the antibodies (rabbit IgG) anti-STAT1 antibody, anti-phosphorylated tyrosine 701 of STAT1 antibody, anti-STAT3 antibody, and anti-phosphorylated tyrosine 705 of STAT3 antibody, diluted as recommended at 1/2000 with Tris-buffered saline with 0.1% Tween 20 with 5% BSA. Antibody binding was detected by horseradish peroxidase-conjugated anti-rabbit IgG antibody diluted at 1/4000 with Tris-buffered saline with 0.1% Tween 20 with 5% BSA, and was revealed using the chemiluminescence system. Protein bands were quantified by densitometry using NIH-Image analysis, and STAT phosphorylation was compared with the total amount of STAT protein. IFN-γ-stimulated Hela cells were used as a positive control for STAT1 phosophorylation.

### Statistical analysis

Data are expressed as the mean value ± standard error of the mean or box plots. The statistical significance of differences between two groups was determined by the Mann–Whitney U test or the Wilcoxon signed rank test. *P *< 0.05 was considered significant.

## Results

### Resistance to IL-10 inhibition of IFN-γ production in RA CD4^+ ^T cells

**Figure 1:**
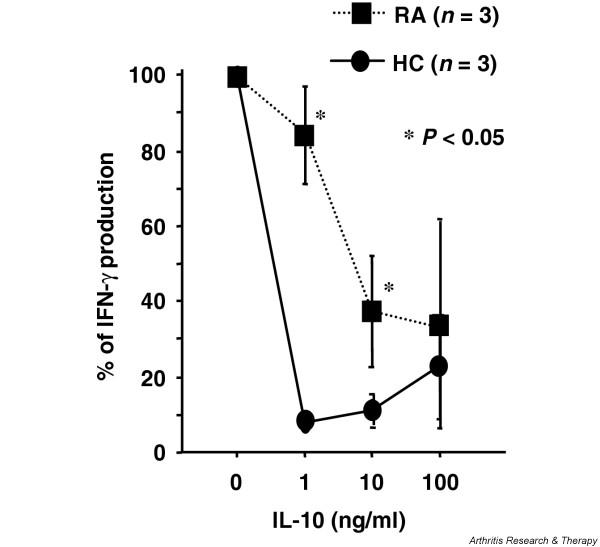
Dose response of IL-10 inhibition of interferon gamma (IFN-γ) production by CD4^+ ^T cells after CD3 and CD28 costimulation in patients with rheumatoid arthritis (RA) and in healthy controls (HC). CD4^+ ^T cells were purified from peripheral blood mononuclear cells of three RA patients and three HC by positive selection with anti-CD4 antibody. CD4^+ ^T cells (5 × 10^5 ^cells in 0.5 ml culture medium with 10% FCS) were stimulated by immobilized anti-CD3 antibody and anti-CD28 antibody in the presence or absence of diluted IL-10 concentrations for 36 hours. Culture supernatants were measured for concentrations of IFN-γ by ELISA. IFN-γ production with IL-10 expressed as % IFN-γ production without IL-10. Values are the mean ± standard error of the mean.

The CD28 costimulatory pathway is crucial for effective antigen-specific T-cell cytokine production, and IL-10 can directly suppress this response by inhibiting CD28 tyrosine phosphorylation and binding of phosphatidylinositol 3-kinase [24]. To evaluate the responsiveness of RA CD4^+ ^T cells to IL-10, purified PB CD4^+ ^T cells from three patients with active RA and from three healthy controls were stimulated by immobilized anti-CD3 antibody and anti-CD28 antibody with or without diluted concentrations of IL-10 for 36 hours, and IFN-γ production was measured by ELISA. As shown in Fig. 1, IFN-γ production by activated normal CD4^+ ^T cells was mostly inhibited at concentrations as low as 1 ng/ml IL-10. However, RA CD4^+ ^T cells were able to produce significant amounts of IFN-γ in the presence of 1 ng/ml IL-10, and the maximal but not complete inhibition by IL-10 was obtained at 10–100 ng/ml.

**Figure 2:**
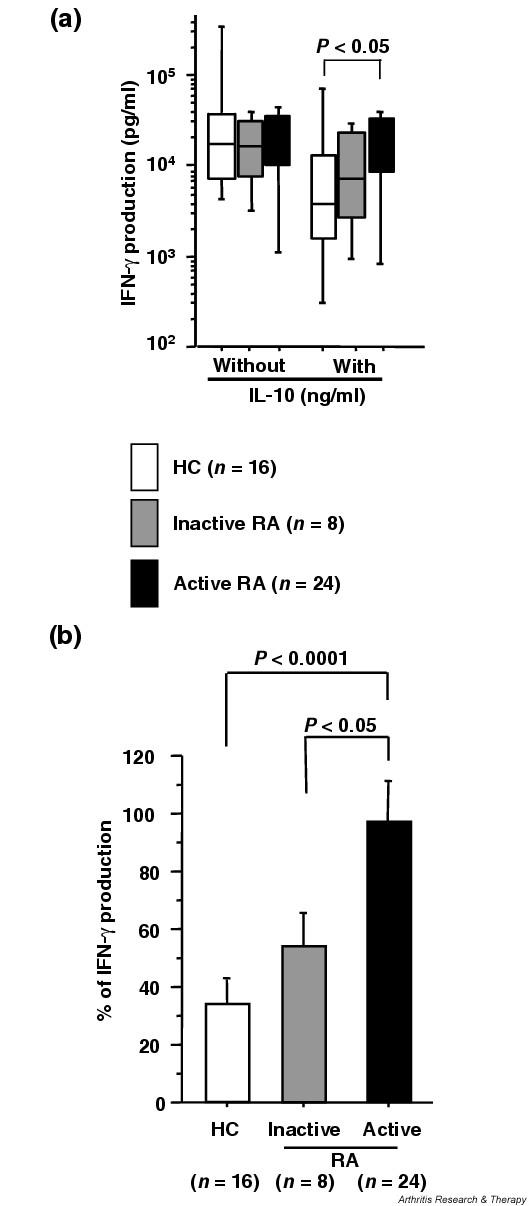
**(a) **Interferon gamma (IFN-γ) production by CD3 and CD28 costimulated CD4^+ ^T cells in the presence of IL-10 in patients with rheumatoid arthritis (RA) and in healthy controls (HC). CD4^+ ^T cells (5 × 10^5 ^cells in 0.5 ml culture medium with 10% FCS) were stimulated by anti-CD3 antibody and anti-CD28 antibody with or without 1 ng/ml IL-10. Concentrations of IFN-γ in culture supernatants were measured in duplicate by ELISA. RA patients were divided into those with active disease (multiple inflammatory joints and CRP level ≥ 10 mg/l) and inactive disease (in remission and CRP level ≤ 4 mg/l). The results are represented as a box plot; upper and lower bars, 90th and 10th percentiles, respectively; upper, center and lower lines of box, 75th, 50th, and 25th percentiles, respectively. **(b) **Percentage of IFN-γ production. IFN-γ production with IL-10 expressed as % IFN-γ production without IL-10. Values are the mean ± standard error of the mean. n, number of samples tested.

We thus compared the levels of IFN-γ production by CD4^+ ^T cells after CD3 and CD28 costimulation in the presence of 1 ng/ml IL-10 in RA patients with active disease (multiple inflammatory joints, CRP level ≥ 10 mg/l) and inactive disease (in remission, CRP level < 10 mg/l) [26] and in healthy controls. There were no statistically significant differences in IFN-γ production without IL-10 among these three groups (Fig. 2a), but the inhibitory effect of IL-10 on IFN-γ production was significantly limited in the active RA group as compared with the inactive RA group and healthy controls (percentage decrease: active RA, 2.9 ± 14.4%; inactive RA, 45.6 ± 14.4%; controls, 65.8 ± 7.9%) (Fig. 2b). As a consequence, CD4^+ ^T cells from active RA patients produced higher levels of IFN-γ in the presence of 1 ng/ml IL-10 than did normal CD4^+ ^T cells (Fig. 2a).

In addition, we compared IL-2 production by CD4^+ ^T cells after CD3 and CD28 costimulation in the presence of IL-10 in active RA patients and in healthy controls. Similarly, IL-2 production was not affected by 1 ng/ml IL-10 in RA patients (percentage decrease, -2.1 ± 13.8%), while it was significantly reduced in healthy controls (61.1 ± 13.7%; *P *< 0.05). Taken together, these results indicate that RA CD4^+ ^T cells become less susceptible to the immunoregulatory effect of IL-10 during the active phase.

### Increased expression of cell surface IL-10R1 on RA CD4^+ ^T cells

**Figure 3:**
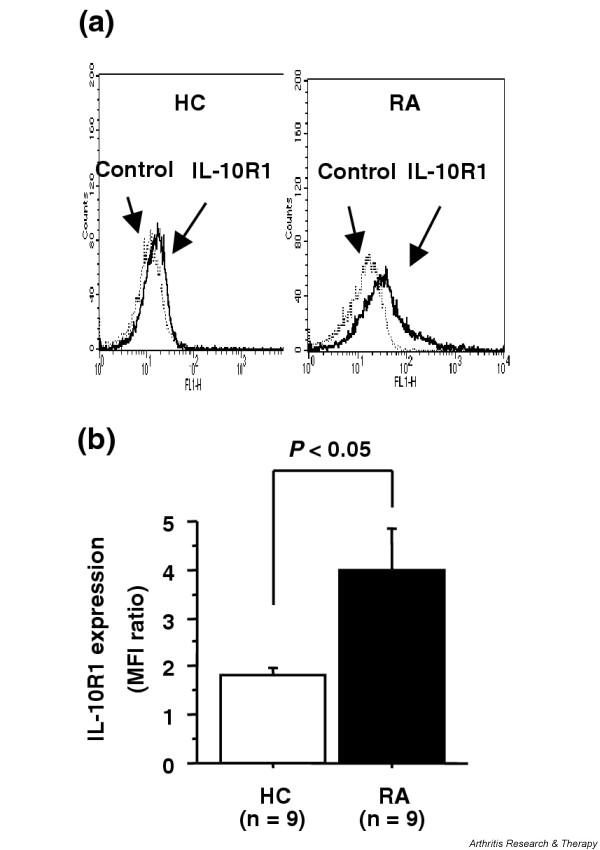
**(a) **Cell surface expression of type 1 interleukin-10 receptor (IL-10R1) on CD4^+ ^T cells from patients with rheumatoid arthritis (RA) and from healthy controls (HC). Peripheral blood mononuclear cells were stained with anti-IL-10R1 antibody or with isotype-matched control antibody, followed by incubation with FITC-conjugated goat anti-mouse IgG1 polyclonal antibody, and were then stained with phycoerythrin-conjugated anti-CD4 mAb. The expression of CD4 and IL-10R1 was determined by flow cytometric analysis. Representative histographic patterns of IL-10R1 expression on CD4^+ ^T cells from RA patients and HC are shown. **(b) **The intensity of IL-10R1 on CD4^+ ^T cells was expressed as the ratio of the mean fluorescence intensity (MFI) of staining with anti-IL-10R1 to control antibody. Values are the mean ± standard error of the mean. n, number of samples tested.

The functional receptor complex of IL-10 consists of two subunits, the primary ligand-binding component IL-10R1 and the accessory component type 2 IL-10R [1]. IL-10R1 expression plays a critical role in cellular responses to IL-10 [29]. To examine whether the resistance to IL-10 inhibition in RA CD4^+ ^T cells was due to limited receptor expression, the cell surface expression of IL-10R1 on PB CD4^+ ^T cells from active RA patients and from healthy controls was determined by flow cytometric analysis. As shown in Fig. 3a,3b, the intensity of IL-10R1 expression on CD4^+ ^T cells was significantly increased in RA patients compared with in healthy controls. These results suggest that the intracellular signal transduction pathway of IL-10 may be impaired in CD4^+ ^T cells of active RA.

### Defective IL-10-mediated STAT3 phosphorylation in RA CD4^+ ^T cells

**Figure 4:**
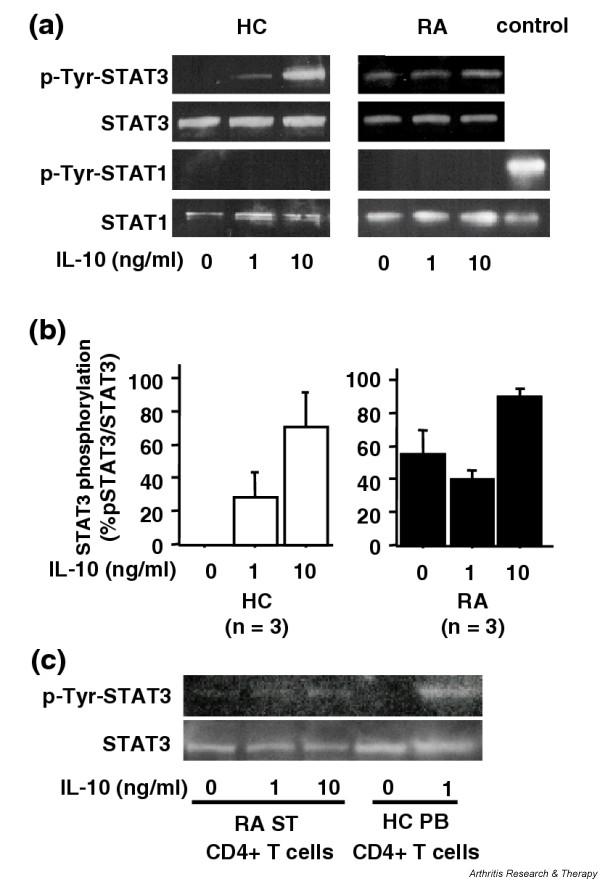
**(a) **IL-10-mediated phosphorylation of signal transducer and activator of transcription (STAT) 1 and STAT3 in CD4^+ ^T cells from patients with rheumatoid arthritis (RA) and from healthy controls (HC). CD4^+ ^T cells (5 × 10^5 ^cells in 0.5 ml culture medium with 10% FCS) were incubated with or without IL-10 (1 and 10 ng/ml) and cells were harvested 20 min later. Whole cell extracts were prepared by placing cells in SDS buffer, and tyrosine phosophorylation (p-Tyr) of STAT1 and STAT3 was detected by western blot analysis. IFN-γ-stimulated Hela cells were used as a positive control for STAT1 phosphorylation. **(b) **Percentage of IL-10-activated STAT3 phosphorylation in CD4^+ ^T cells from RA patients and from HC. Protein bands were quantified by densitometry using NIH-Image analysis, and STAT3 phosphorylation was expressed as % total STAT3 protein. **(c) **STAT3 phosphorylation in ST CD4^+ ^T cells from RA patients. Representative results of STAT3 phosphorylation in CD4^+ ^T cells from three synovial tissue samples of RA patients and three peripheral blood samples of HC are shown. Values are the mean ± standard error of the mean. n, number of samples tested.

The interaction of IL-10R with IL-10 induces tyrosine phosphorylation and activation of the latent transcription factors STAT1 and STAT3 [3]. Macrophage-specific STAT3-deficient mice demonstrated that STAT3 plays a dominant role in IL-10-mediated anti-inflammatory responses [5], which has recently been confirmed in human macrophages by studies of dominant-negative STAT3 overexpression [30]. The induction of STAT1 and STAT3 phosphorylation by IL-10 in PB CD4^+ ^T cells from active RA patients and from healthy controls was examined using western blotting. STAT3 phosphorylation was dose-dependently induced after IL-10 activation for 20 min in normal CD4^+ ^T cells (Fig. 4a,4b). In contrast, STAT3 was phosphorylated in freshly isolated PB CD4^+ ^cells from RA patients and this STAT3 phosphorylation was detectable for up to 6 hours. STAT3 phosphorylation was augmented only when activated by as much as 10 ng/ml IL-10. Both sustained STAT3 phosphorylation and defective IL-10-induced STAT3 phosphorylation were found in RA ST CD4^+ ^T cells (Fig. 4c). On the other hand, IL-10-induced STAT1 phosphorylation was not detected in either RA CD4^+ ^T cells or normal CD4^+ ^T cells (Fig. 4a). These results indicate that STAT3 is the major IL-10-activated STAT in CD4^+ ^T cells, and IL-10-induced STAT3 activation may be diminished in active RA, in association with sustained STAT3 phosphorylation.

### IL-6-mediated STAT3 phosphorylation and inhibition of IL-10 effect in normal CD4^+ ^T cells

**Figure 5:**
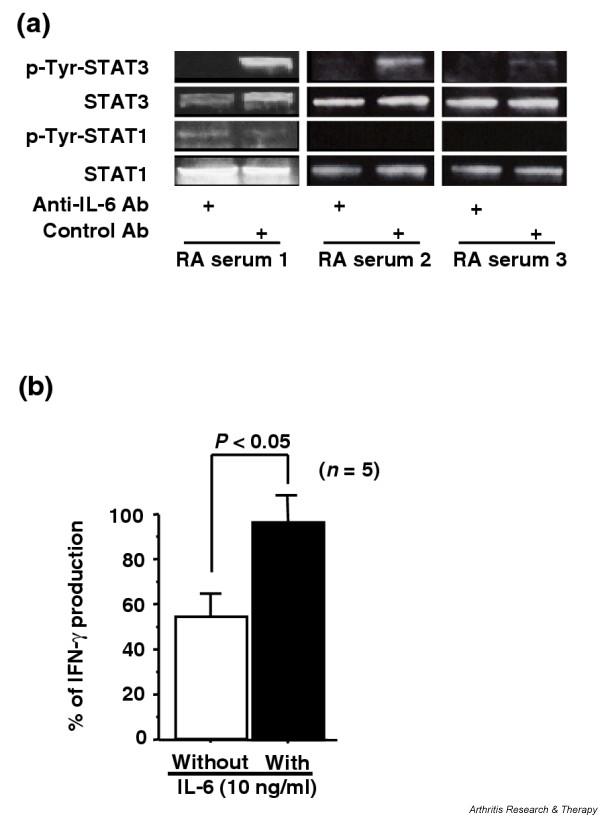
**(a) **Activation of signal transducer and activator of transcription (STAT) 3 in normal CD4^+ ^T cells by serum IL-6 from patients with rheumatoid arthritis (RA). CD4^+ ^T cells from healthy controls (5 × 10^5 ^cells in 0.5 ml culture medium) were stimulated by 30% RA serum in the presence of neutralizing anti-IL-6 antibody (Ab) (40 μg/ml) or of control antibody (40 μg/ml) for 20 min. Phosophorylation of STAT1 and STAT3 was detected by western blot analysis.**(b) **Effect of IL-6 pretreatment on IL-10 inhibition of IFN-γ production by CD4^+ ^T cells. CD4^+ ^T cells (5 × 10^5 ^cells in 0.5 ml culture medium with 10% FCS) were incubated with or without IL-6 (10 ng/ml) for 36 hours, and were then stimulated by anti-CD3 antibody and anti-CD28 antibody in the presence or absence of IL-10 (1 ng/ml) for 36 hours. Concentrations of IFN-γ in culture supernatants were measured in duplicate by ELISA. IFN-γ production with IL-10 was expressed as % IFN-γ production without IL-10. Values are the mean ± standard error of the mean. n, number of samples tested. P-Tyr, tyrosine phosophorylation.

STAT3 is activated by many cytokines and growth factors such as the IL-6 family of cytokines (IL-6, IL-11, leukemia inhibitory factor, and oncostatin M), platelet-derived growth factor, and epidermal growth factor, in addition to IL-10 [4], but previous studies have demonstrated that IL-6 is the major factor in RA synovial fluid that induces constitutive activation of STAT3 in mononuclear cells [31]. Since IL-6 is also abundant in sera of active RA patients, frequently detected at > 1 ng/ml [27], we examined whether persistent exposure of CD4^+ ^T cells to high concentrations of IL-6 in the blood circulation was responsible for their sustained STAT3 activation and resistance to IL-10 inhibition in active RA. Both STAT1 and STAT3 phosphorylation was activated by IL-6 in normal CD4^+ ^T cells (data not shown), in agreement with previous observations [4]. Normal CD4^+ ^T cells were thus incubated for 20 min with culture medium containing 30% serum from active RA patients and neutralizing anti-IL-6 antibody or control antibody, and STAT phosphorylation was examined by western blot analysis. RA serum was able to induce tyrosine phosphorylation of STAT3 but not STAT1, and this STAT3 activation was mostly abolished by neutralization of IL-6 activity (Fig. 5a). These results indicate that IL-6 is the dominant STAT3-activating factor contained in sera of active RA patients. The lack of STAT1 activation by RA serum suggests that much higher concentrations of IL-6 may be required for STAT1 activation as compared with STAT3 activation, or that inhibitors of STAT1 signaling may be present in RA serum.

We next examined whether IL-6 could suppress the effect of IL-10 to inhibit IFN-γ production by CD4^+ ^T cells. After preincubation with or without 10 ng/ml IL-6 for 36 hours, normal CD4^+ ^T cells were stimulated by CD3 and CD28 costimulation in the presence or absence of 1 ng/ml IL-10 for 36 hours, and the IFN-γ production was measured by ELISA. IL-6 pretreatment of normal cells reduced IL-10-mediated inhibition of IFN-γ production (Fig. 5b), indicating that high concentrations of IL-6 could modulate T-cell responsiveness to IL-10. Taken together, these findings suggest that persistent exposure to serum IL-6 may have a role in both the induction of STAT3 activation and the resistance to the inhibitory effect of IL-10 in RA CD4^+ ^T cells.

### High expression of SOCS1 mRNA in RA CD4^+ ^T cells

**Figure 6:**
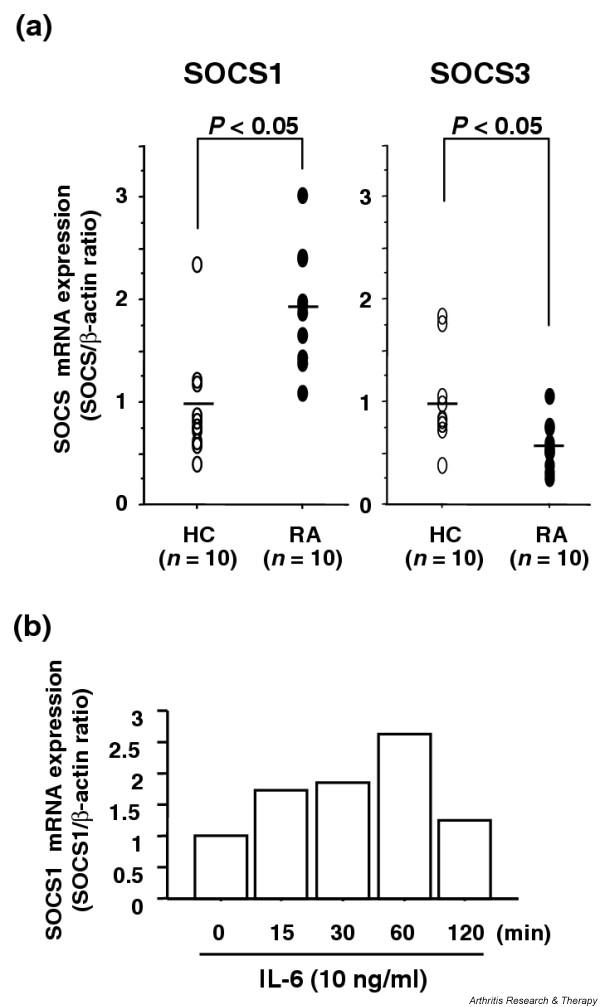
**(a) **The mRNA expression of SOCS1 and SOCS3 in CD4^+ ^T cells from patients with RA and healthy controls (HC). Total cellular RNA was extracted from freshly isolated CD4^+ ^T cells and mRNA expression of SOCS1 and SOCS3 was analyzed by real time-PCR as described in Patients and Methods. Levels of SOCS1 and SOCS3 mRNAs were normalized relative to β-actin expression. Values are the mean ± SEM. n = number of samples tested. **(b) **Kinetics of IL-6-induced SOCS1 mRNA expression in normal CD4^+ ^T cells. CD4^+ ^T cells from HC (5 × 10^5 ^cells in 0.5 ml of culture medium with 10% FCS) were stimulated with IL-6 (10 ng/ml) and SOCS1 mRNA expression was determined at the indicated time after stimulation.

IL-6 induces two potent inhibitors of JAKs (SOCS1 and SOCS3 proteins) that not only act as mediators of negative feedback inhibition, but also play a major role in crosstalk inhibition by opposing other cytokine-signaling pathways [7]. SOCS3 has recently been shown to specifically inhibit STAT3 activation induced by IL-6 but not by IL-10, thereby regulating the divergent action of IL-6 and IL-10 [8,9]. On the contrary, SOCS1 is able to partially inhibit IL-10-mediated STAT3 activation and cellular responses, as well as IFN-γ-mediated STAT1 activation [32]. To determine whether SOCSs were involved in the defective IL-10-induced STAT3 activation of RA CD4^+ ^T cells, the levels of SOCS1 and SOCS3 mRNA expression in PB CD4^+ ^T cells from active RA patients and from healthy controls were compared by semiquantitative real-time PCR. The RA CD4^+ ^T cells contained higher levels of SOCS1 but lower levels of SOCS3 transcripts than did control CD4^+ ^T cells (Fig. 6a). Constitutive expression of SOCS1 mRNA in RA CD4^+ ^T cells was comparable with the expression in normal CD4^+ ^T cells stimulated by 10 ng/ml IL-6 (Fig. 6b), supporting its functional significance. Defective IL-10-induced STAT3 activation therefore appears to be due at least in part to an abundance of SOCS1 in RA CD4^+ ^T cells.

## Discussion

CD4^+ ^T cells orchestrate the Th1-type cell-mediated immune response in RA [22]. Activated CD4^+ ^T cells stimulate macrophages, synovial fibroblasts, B cells, and osteoclasts through the expression of cell surface molecules and Th1 cytokines, thereby contributing to both the chronic inflammation and the joint destruction. CD4^+ ^T cells require two signals to be activated; antigen receptor occupancy and CD28-mediated costimulation. In the ST lesion, the CD28 ligands, both CD80 and CD86, together with MHC class II antigens, are substantially expressed by antigen-presenting cells such as macrophages and dendritic cells [18-20]. The significance of CD28 engagement in the T-cell-mediated disease process has recently been proven by the clinical efficacy of its blocker cytotoxic T-cell-associated antigen 4 (CD152)-IgG in RA patients [23].

IL-10 plays a predominant role in limiting immune and inflammatory responses by regulating the function of both macrophages and Th1 cells [1]. IL-10 inhibits the tyrosine phosphorylation of the CD28 molecule and the subsequent phosphatidylinositol 3-kinase binding in T cells, and thereby directly acts on T cells [24]. In the present study, we found that PB CD4^+ ^T cells from patients with active RA, in the presence of IL-10, are able to produce higher levels of IFN-γ after CD3 and CD28 costimulation than normal CD4^+ ^T cells. Despite high-level IL-10R1 expression and constitutive STAT3 activation, IL-10-induced tyrosine phosphorylation of STAT3 is suppressed in RA CD4^+ ^T cells, in contrast to normal CD4^+ ^T cells, where STAT3 phosphorylation is dose-dependently inducible by IL-10. Serum IL-6 from RA patients induces STAT3 but not STAT1 phosphorylation in normal CD4^+ ^T cells, and exogenous IL-6 induces the resistance to IL-10 inhibition of IFN-γ production. RA CD4^+ ^T cells contain higher levels of SOCS1 but contain lower levels of SOCS3 transcripts in comparison with normal CD4^+ ^T cells. These findings indicate that CD4^+ ^T cells become resistant to the inhibitory effect of IL-10 before migration into the inflamed ST, and suggest that this resistance may be attributable to impaired IL-10-dependent STAT3 activation, in association with sustained STAT3 phosphorylation and SOCS1 induction.

IL-10-mediated inhibition of CD4^+ ^T-cell cytokine production is principally dependent on its inhibition of macrophage antigen-presenting cell function [1]. However, this indirect inhibitory effect is thought to be restricted at the site of T-cell activation in RA, because macrophages in the ST express high levels of cytokines, CD80 and CD86 molecules, and MHC class II antigens [10,18-20]. More recently, IL-10 has been shown to induce the antigen-specific T-cell unresponsiveness by inhibiting CD28 tyrosine phosphorylation [33]. This direct effect also may be limited in active RA patients, because their PB CD4^+ ^T cells showed a defective IL-10 inhibition of CD28-costimulated production of both IFN-γ and IL-2.

Numerous cytokines, both proinflammatory and anti-inflammatory, have been detected in the ST of RA, and the balance between these opposing cytokine activities regulates disease severity [10]. Endogenous IL-10, produced mainly by macrophages and T cells, inhibits proinflammatory cytokine production by ST cells [12]. However, this regulatory activity seems to be restricted during chronic inflammation. The activation of both the extracellular stimulus-regulated kinase and p38 kinase pathways, induced by TNF-α and IL-1, inhibits the Jak1–STAT3 signaling pathway shared by IL-10 and IL-6 in adhered macrophages [13]. More importantly, IL-10-mediated STAT3 activation is mostly undetectable in RA synovial macrophages. This impaired IL-10 signaling is probably induced by chronic exposure to immune complexes *in vivo*, because both cell surface IL-10R1 expression and IL-10-induced Jak1 activation are suppressed in IFN-γ-primed macrophages by a protein kinase C-dependent pathway following ligation of the IgG Fc gamma receptor [14]. Furthermore, dendritic cells from RA synovial fluids are resistant to the immunoregulatory effect of IL-10 due to decreased transport of intracellular IL-10R1 in the presence of proinflammatory cytokine stimuli such as TNF-α, IL-1, and granulocyte–macrophage colony-stimulating factor [15]. We have demonstrated that the resistance of RA CD4^+ ^T cells to IL-10 may be associated with defective IL-10-dependent STAT3 activation, but not with IL-10R1 expression. Inhibitory effects of IL-10 on these inflammatory cell types are therefore differentially modulated at the signal transduction level under the inflammatory environment in RA.

In association with impaired IL-10-mediated STAT3 activation, STAT3 was found to be tyrosine phosphorylated persistently (up to 6 hours) in freshly isolated PB and ST CD4^+ ^T cells from RA patients. STAT3 is activated by a variety of cytokines, notably the IL-6 family of cytokines (e.g. IL-6, IL-11, leukemia inhibitory factor, and oncostatin M) and growth factors, in addition to IL-10 [4]. Of these cytokines, IL-6 plays a predominant role in eliciting a systemic reaction such as the acute phase response in active RA, due mainly to its abundance in the blood circulation [27]. Consistent with this notion, IL-6 was the major STAT3-activating factor contained in the serum of active RA patients, and the responsiveness to IL-10 was suppressed in normal CD4^+ ^T cells after 36 hours of incubation with IL-6. These results suggest that both the sustained STAT3 activation and the resistance to IL-10 inhibition found in RA CD4^+ ^T cells may be induced after chronic exposure *in vivo *to high concentrations of serum IL-6. However, it is also possible that STAT3 activity could be constitutively induced in CD4^+ ^T cells by their own IL-10 secretion, leading to the loss of sensitivity to exogenous IL-10, because RA CD4^+ ^T cells in the ST are capable of producing significant levels of IL-10 [34].

CD4^+ ^T cells isolated from the ST of RA also showed a defect in the IL-10-induced STAT3 signaling pathway. It is most probable that the resistance of CD4^+ ^T cells to IL-10 can be even augmented after migration into the inflamed ST, because IL-6 is highly concentrated compared with the blood level [27]. In addition, the involvement of other essential proinflammatory cytokines in this process was suggested by our preliminary experiments demonstrating that IL-10-mediated IFN-γ inhibition in CD4^+ ^T cells was reduced by pretreatment with IL-1β and TNF-α, although less effectively than by IL-6 (data not shown). Furthermore, IFN-γ and IL-10 produced by CD4^+ ^T cells themselves could be responsible for impaired IL-10 signaling in the ST, because T-cell infiltrates produce both cytokines [34,35]. In an autocrine fashion, IL-10 may persistently stimulate STAT3 activation and IFN-γ can induce SOCS1 protein as a crosstalk inhibitor of IL-10 signaling [32]. The T-cell-inhibitory effect of IL-10 may therefore be modulated complicatedly upon exposure to an inflammatory environment in RA joints, where many cytokines are present substantially [10].

STAT3 activation has been implicated in the pathogenesis of RA. Active STAT3 is constitutively expressed in synovial fluid mononuclear cells from RA patients [36]. IL-6 is the major STAT3-activating factor present in synovial fluid, which has a crucial role in the activation of monocyte functions such as gene expression of the Fc gamma receptor type I and type III and of HLA-DR [31]. More recently, high levels of activated STAT3, thought to be induced mainly by IL-6, have been detected in the ST, and STAT3 activation has been shown to be involved in synovial fibroblast proliferation and IL-6 production [37]. In this regard, STAT3 is critical in the survival and expansion of growth factor-dependent synovial fibroblasts [38]. Furthermore, the significance of persistent STAT3 signaling in Th1-cell-dominated autoimmune arthritis has been suggested by studies of the *gp130*^*F*759/*F*759 ^mice, in which the Src homology phosphatase-2 binding site of gp130 (the transmembrane glycoprotein β subunit of the IL-6 family cytokine receptor), tyrosine 759, was mutated to phenylalanine [39]. In the *gp130*^*F*759/*F*759 ^mice, T cells, particularly the CD4^+ ^T-cell subset, are chronically activated and resistant to activation-induced cell death through gp130-mediated STAT3 activation.

The longevity of cytokine signals transduced by the JAK–STAT pathway is regulated by the SOCS family proteins [7]. We found that CD4^+ ^T cells from patients with active RA expressed higher levels of SOCS1, but lower levels of SOCS3, compared with normal CD4^+ ^T cells. SOCS1 prevents activation of JAK by directly binding to JAK, and SOCS3 inhibits the action of JAK by binding to the Src homology phosphatase-2-binding domain of receptors such as gp130 [40]. SOCS1 and SOCS3 are induced by various cytokines, including IL-6 and IL-10, as mediators of negative feedback and crosstalk inhibition [7]. Recent studies with mice lacking SOCS3 or SOCS1 revealed that SOCS3 is a negative regulator of IL-6 signaling but not of IL-10 signaling. Studies of conditional SOCS3-deficient mice have shown that SOCS3 deficiency, but not SOCS1 deficiency, results in sustained activation of STAT3 in response to IL-6 [8,41]. The analysis of SOCS3-deficient macrophages has indicated that SOCS3 is a crucial inhibitor of the IL-6-induced transcriptional response [42]. However, SOCS3 is dispensable for both the negative feedback inhibition and the immunoregulatory action of IL-10 in macrophages [41]. On the contrary, SOCS1 was found to directly inhibit IL-10-mediated signaling [43]. Increased SOCS1 expression in RA CD4^+ ^T cells may therefore be associated with both the impaired responsiveness to IL-10 and to IL-10-mediated STAT3 activation, and defective SOCS3 expression may be responsible for persistent STAT3 activation in response to serum IL-6.

There is a possibility that SOCS1 induction may be associated with the ability of CD4^+ ^T cells to produce IFN-γ, because CD4^+ ^T cells from active RA could produce high levels of IFN-γ in the presence of IL-10, and because IFN-γ has been known as a potent inducer of SOCS1 [32]. It is of interest in this regard to indicate that polarized Th1 and Th2 cells express high levels of SOCS1 and SOCS3 mRNA, respectively [44]. IL-12-induced STAT4 activation is inhibited by SOCS3 induction in Th2 cells, whereas IL-4-induced STAT6 signaling is diminished by SOCS1 induction in Th1 cells. SOCS1 and SOCS3 may thus have important roles as Th1-specific and Th2-specific, mutually exclusive, cross-talk repressors of the IL-4–STAT6 and the IL-12–STAT4 signaling pathways, respectively. Consistent with this notion, PB T cells from patients with allergic diseases significantly express high levels of SOCS3 transcripts, and the SOCS3 expression correlates well with serum IgE levels and disease pathology [45]. Higher SOCS1 expression with lower SOCS3 expression in PB CD4^+ ^T cells from RA patients, compared with healthy controls, is therefore probably consistent with their systemic bias towards a Th1 phenotype, as has previously been demonstrated [46-49].

## Conclusion

CD4^+ ^T cells from active RA patients are characterized by their resistance to IL-10 inhibition of IFN-γ production, due to constitutive STAT3 phosphorylation and impaired IL-10-mediated STAT3 activation. The defective STAT3 signaling is possibly associated with SOCS1 predominance over SOCS3. These abnormalities in active RA are thought to be induced mainly after chronic exposure to high concentrations of IL-6. The limited efficacy of IL-10 treatment of RA patients [50] may be explained in part by the unresponsiveness to IL-10 of inflammatory cells, including T cells. On the contrary, the therapeutic efficacy of anti-IL-6 receptor antibody has been reported in RA patients [51], and one of the effects of this therapy may be to normalize T cells through the inhibition of IL-6-dependent STAT3 activation. More specific therapy targeting STAT3 activation will be awaited; for example, the induction of the SOCS3 gene, the efficacy of which has been demonstrated in animal models [37].

## Abbreviations

BSA = bovine serum albumin; CRP = C-reactive protein; ELISA = enzyme-linked immunosorbent assay; Fc = crystallazibe fragment; FCS = fetal calf serum; FITC = fluorescein isothiocyanate; IFN-γ = interferon gamma; IL = interleukin; IL-10R = interleukin-10 receptor; IL-10R1 = type 1 interleukin-10 receptor; JAK = Janus kinase; mAb = monoclonal antibody; MHC = major histocompatibility complex; PB = peripheral blood; PBMC = peripheral blood mononuclear cells; PBS = phosphate-buffered saline; PCR = polymerase chain reaction; RA = rheumatoid arthritis; SOXS = suppressor of cytokine signaling; ST = synovial tissue; STAT = signal transducer and activator of transcription; Th = T helper cells; TNF-α = tumor necrosis factor alpha.

## Competing interests

The author(s) declare that they have no competing interests.

## Authors' contributions

Jiro Yamana was responsible for the experiments and data analysis and wrote the report. Masahiro Yamamura was responsible for the planning of the research and wrote up the manuscript. Akira Okamoto, Tetsushi Aita, Mitsuhiro Iwahashi, and Katsue Sunahori assisted the experiments. Hirofumi Makino critically read the manuscript.
